# On the accuracy of displacement-based wave intensity analysis: Effect of vessel wall viscoelasticity and nonlinearity

**DOI:** 10.1371/journal.pone.0224390

**Published:** 2019-11-01

**Authors:** Jingyi Kang, Arian Aghilinejad, Niema M. Pahlevan

**Affiliations:** 1 Department of Aerospace and Mechanical Engineering, University of Southern California, Los Angeles, CA, United States of America; 2 Keck School of Medicine, University of Southern California, Los Angeles, CA, United States of America; Texas A&M University System, UNITED STATES

## Abstract

Recent studies showed that wave intensity analysis (WIA) provides clinically valuable information about local and global cardiovascular function. Wave intensity (WI) is computed as the product of the pressure change and the velocity change during short time intervals. The major limitation of WIA in clinical practice is the need for invasive pressure measurement. Since vessel wall displacement can be measured non-invasively, the usage of WI will be expanded if the vessel wall dilation is used instead of pressure in derivation of WI waveform. Our goal in this study is to investigate the agreement between wall displacement-based WI and the pressure-based WI for different vessel wall models including linear elastic, nonlinear and viscoelastic cases. The arbitrary Eulerian Lagrangian finite element method is employed to solve the coupled fluid-structure interaction (FSI). Our computational models also include two types of vascular disease-related cases with geometrical irregularities, aneurysm and stenosis. Our results show that for vessels with linear elastic wall, the displacement-based WI is almost identical to the pressure-based WI. The existence of vessel irregularities does not impact the accuracy of displacement-based WI. However, in a viscoelastic wall where there is a phase difference between pressure and vessel wall dilation, displacement-based WI deviated from pressure-based WI. The error associated with this phase difference increased nonlinearly with increasing viscosity. This results in a maximum error of 6.8% and 7.13% for a regular viscoelastic vessel wall and an irregular viscoelastic vessel wall, respectively. A separate analysis has also been performed on the agreement of backward and forward running waves extracted from a decomposition of the displacement-based and pressure-based WI. Our findings suggest that displacement-based WI is a reliable method of WIA for large central arteries that do not show viscoelastic behaviors. This can be clinically significant since the required information can be measured non-invasively.

## Introduction

It is well accepted that arterial waveform analysis provides clinically valuable information about local and global cardiovascular function. Due to the complex dynamic interactions of hemodynamic waves (e.g., pressure and flow), extracting clinically reliable information about the state of health or disease remains a significant challenge to modern medicine [1]. Pressure and flow pulse waves in large arteries are composed of forward running waves originated by the ventricle and backward running (reflected) waves created by the vascular network. Understanding the physics of these forward and backward waves is necessary for extracting physiological information relevant to clinical practice. Several methods such as Fourier (impedance) methods and wave intensity analysis (WIA) have been introduced for decomposing hemodynamic waves in the arterial system into their forward and backward components. The Fourier analysis method (impedance method), in particular, relies on the normal periodicity of the cardiac cycle to compute the hemodynamic impedance in the frequency domain [2]. Therefore, this method is not useful for many applications in which the timeline of certain events is important [3]. This method additionally assumes a linear relation between the pressure and flow rate at each harmonic frequency.

WIA was introduced by Parker and Jones as an alternative time domain method for separating forward and backward waves based on the method of characteristics—without needing to assume linearity or periodicity since it relies on the solution of conservation equations in the time domain [4]. In this method, wave intensity (WI) is computed as the product of the blood pressure change and the velocity change during short time intervals [5]. A positive value of WI indicates that forward-traveling waves predominate while a negative value indicates that backward-traveling waves predominate. WIA can also be used to compute physiologically relevant indices such as local pulse wave velocity [6]. Since WI is in units of power per area (an index of energy per unit area carried by waves), the WIA method can determine wave directionality as well as the magnitude of the energy transferred by the waves [5]. WIA has been used in hemodynamic studies throughout the cardiovascular circulatory network including the ascending aorta [7], carotid arteries [8], pulmonary circulation [9], and coronary arteries [10], and has demonstrated promising abilities in providing clinical insights useful for early diagnosis in cardiovascular diseases.

Although the WIA can provide valuable information in clinical study and cardiovascular research, this method requires simultaneous measurement of both pressure and flow waves at the same location (which makes it clinically challenging). In current clinical practice, simultaneous pressure and velocity measurements can be obtained invasively using sensor-tipped guidewires [11] (e.g., ComboWire, Volcano Inc). In general, invasive procedures are more expensive, may cause patient discomfort/pain, and have a higher risk of clinical complications. Therefore, non-invasive evaluation of WI [12] is prudent to expand the clinical utility and efficacy of this method. Already, blood velocity can be easily measured non-invasively with imaging modalities such as echocardiograms and phase contrast (PC) magnetic resonance imaging (MRI). The non-invasive version of WIA can use the diameter change of the vessel wall instead of invasively measured blood pressure waves. This technique has been explored in order to evaluate important hemodynamic indices such as pulse wave velocity [13] or WI [14]. As an example, Khir *et al*. have shown the separation of WI into its forward and backward directions using the non-invasively measured diameter of a flexible tube’s wall and the corresponding flow velocity [12]. However, it is well-known that blood vessels exhibit complex and viscoelastic properties that are attributed to the smooth muscle cells. This viscoelasticity may introduce a phase difference between the pressure and wall deformation [15] which may subsequently introduce an error in the non-invasive evaluation of WI.

Our goal in this study is to investigate the agreement between non-invasive wall displacement-based WI and the pressure-based WI. Computational fluid dynamics (CFD) with Fluid-Solid interaction (FSI) has been employed to simulate pressure and flow waves in various vessel models. In the mammalian cardiovascular system, arteries can be roughly divided into two types: i) proximal large arteries that show linear elastic behavior (under normal conditions) and ii) distal small arteries that demonstrate viscoelastic and nonlinear (stress-strain) behavior [16]. In this study, the wall models have included linearly elastic, non-linear, and viscoelastic arterial walls. Additionally, we have examined the accuracy of displacement-based WIA for cases with downstream geometrical irregularities such as aneurysm (relevant to abdominal aortic aneurysm) and stenosis (relevant to atherosclerosis). The systematic research work conducted here provides the fundamental groundwork for utilizing the non-invasive displacement-based WIA technique to measure the clinically relevant quantities such as WI.

## Materials and methods

### Physical model

**Fig 1:**
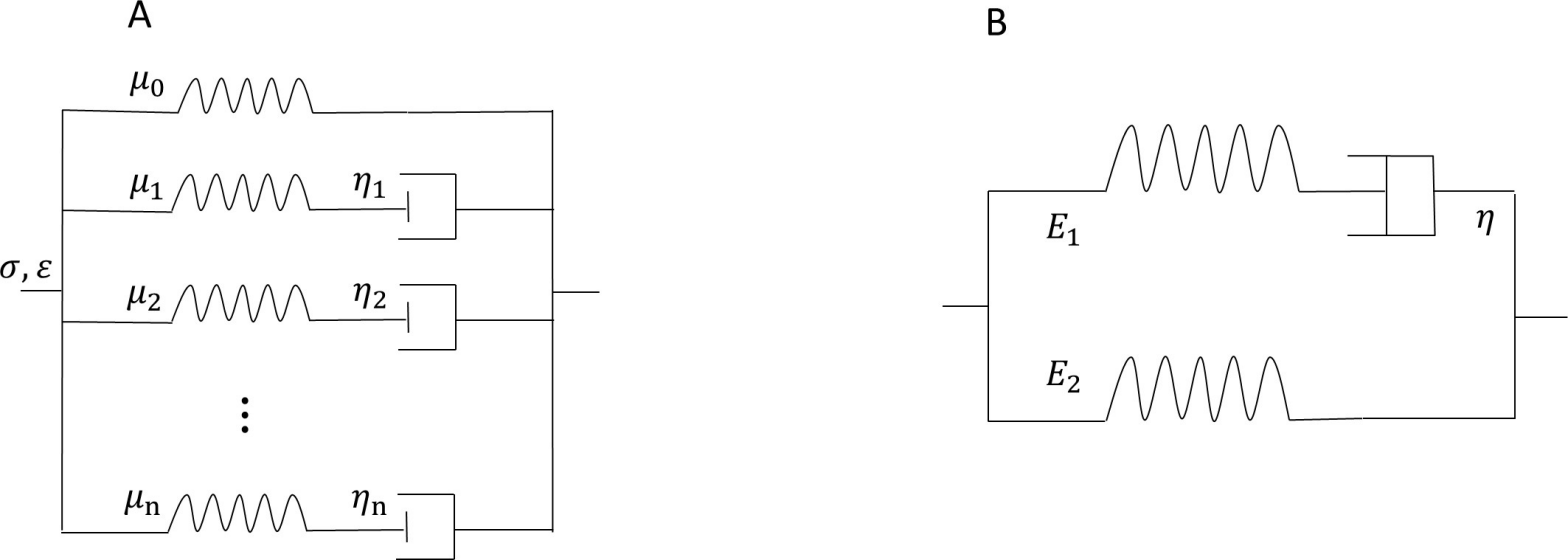
Schematic representation of the Generalized Maxwell model on the left (A) and the standard solid viscoelastic model on the right (B).

In order to comprehensively investigate the WI in different arteries, we have considered two cases: (1) vessel walls with no viscoelastic behavior representing large central arteries such as aorta and carotid artery, and (2) vessel walls with considerable degree of viscoelasticity representing the small and muscular arteries. The generalized Maxwell model was considered for the viscoelastic wall (Fig 1). In this model, a linear spring *μ*_*0*_ is connected in parallel to *n* terms of Maxwell elements, where each element is composed of a linear spring and a viscous dashpot [17]. In the *i*th Maxwell element, the spring has an elastic modulus *μ*_*i*_ and the dashpot has a viscous coefficient *η*_*i*_. The *σ* and *ε* in Fig 1 are the stress and strain for the whole system, respectively [18]. Specifically, we utilized the first two terms of generalized Maxwell viscoelastic model which consisted of an elastic part with Young’s modulus *E*_*1*_, the viscous part with Young’s modulus *E*_*2*_, and the damping coefficient of the dashpot *η*.

**Fig 2:**
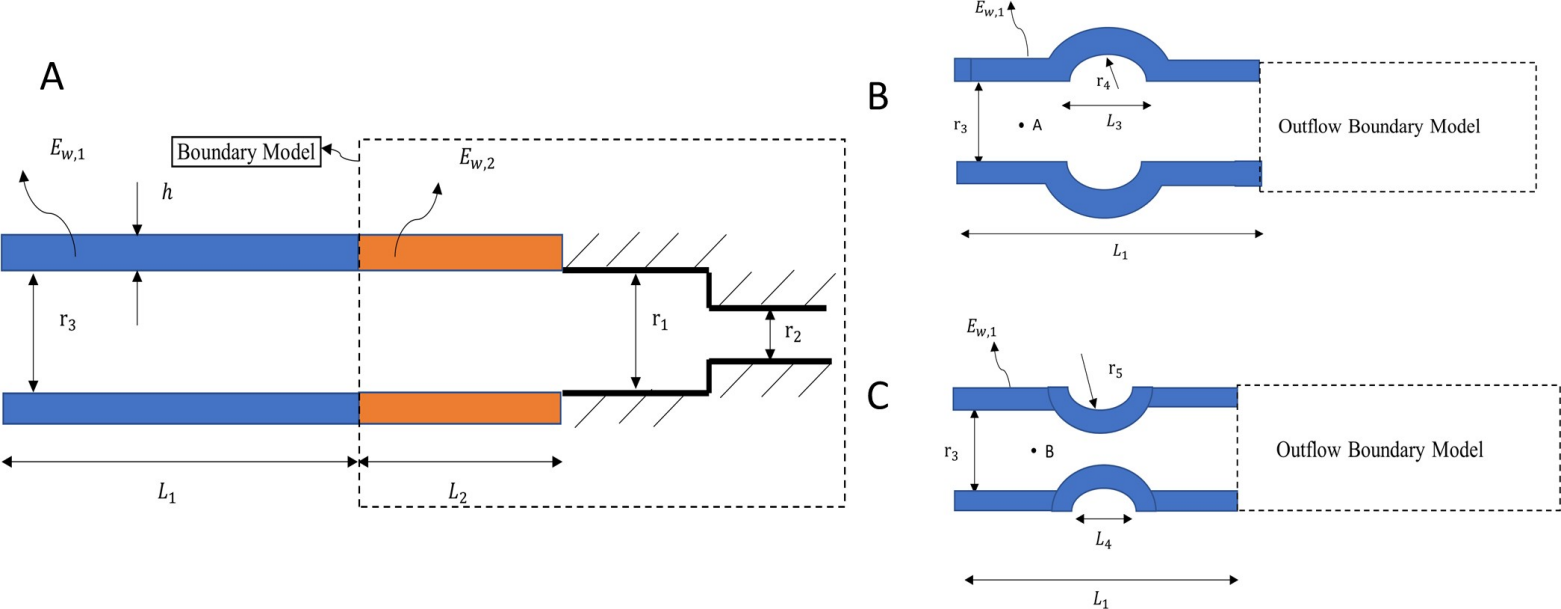
(A) Straight vessel connected to the outflow boundary condition, (B) Abdominal aortic aneurysm and (C) Stenosis cases connected to the same outflow boundary to capture the effect of truncated downstream vasculature.

The extension tube boundary model [19] was used as the outflow boundary model to acquire physiologically relevant solutions for pressure, diameter, and velocity waves. This outflow boundary model can capture the compliance, resistance, and wave reflections of the downstream vasculature [19], and it consists of a straight elastic tube connected to a rigid contraction tube (Fig 2). We first studied a simple case of a straight vessel with uniform thickness and a circular cross-section (Fig 2A). Following this case, vessel models with geometrical irregularities (sudden dilation or constriction) were investigated to evaluate the accuracy of the displacement-based WIA in the presence of vascular diseases such as stenosis and aneurysm (Fig 2B and 2C). The same outflow boundary model was used in all these cases.

The geometrical and outflow parameters of the vessel models (see Fig 2) are provided in Table 1. The geometric data, such as the radius and the length of the bulge in aneurysm and stenosis cases, are chosen within the average psychological range [20]. To characterize the viscoelasticity of the vessel wall, the relaxation modulus (*E*(*t*)) is utilized in response to strain loading for the Maxwell-type standard solid viscoelastic material model. This dynamic modulus includes the effects of E_1_ and E_2_ as shown in Fig 1B. In all simulations, blood is assumed to be a incompressible and Newtonian fluid with the properties shown in Table 1.

**Table 1:** Parameters employed for computational modeling.

Material Properties		
Symbol	Description	Value
*E*_*w*,1_	Young’s modulus of the straight vessel	1×10^5^ Pa
*E*_*w*,2_	Young’s modulus of the boundary model	2×10^5^ Pa
*h*	Wall thickness of the elastic boundary tube	0.1 cm
*L*_*2*_	Length of the elastic boundary tube	10 cm
*L*_*1*_	Length of the straight vessel	20 cm
*r*_*3*_	Internal radius of the straight vessel	1 cm
*γ*	Contraction ratio of the rigid boundary tube	0.5
*υ*	Poisson ratio of straight vessel and elastic boundary	0.45
*ρ*_*s*_	Density of straight vessel and elastic boundary	1 g/cm^3^
*μ*	Viscosity of the fluid	0.0051 Pa·s
*ρ*_*f*_	Density of the fluid	1050 kg/m^3^
*η*	Viscosity of the aorta wall	1000–12000 Pa·s
**Aneurysm**		
*L*_*3*_	Length of the bulge for aneurysm case	4 cm
*r*_*4*_	Internal radius of the bulge for aneurysm case	1.865 cm
**Stenosis**		
*L*_*4*_	Length of the bulge for stenosis case	2 cm
*r*_*5*_	Internal radius of the bulge for stenosis case	1.55 cm

### Theoretical considerations

Prior to introducing our computational model, it is beneficial to consider an analytical model for vessel wall displacement with standard solid viscoelastic model. The constitutive relationship (stress-strain) can be considered as [15]

$$\sigma (t)+a_{1}\dot{\sigma }(t)=m\varepsilon (t)+b_{1}\dot{\varepsilon }(t)),$$ (1)

where *a*_*1*_, *m*, *b*_*1*_ are constants and *a*_*1*_ = *η / E*_*1*_, *m* = *E*_*2*_, *b*_*1*_ = *η (E*_*1+*_ *E*_*2*_*) / E*_*1*_.

The relaxation modulus *E(t)*, in response to strain loading for Maxwell-type standard solid viscoelastic material model, is then given by

$$E(t)=E_{\infty }+{{(E_{0}-E}_{\infty })e}^{\frac{-t}{\tau_{0}}},$$ (2)

where *E*_∞_ = *E*_2_, *E*_*0*_ = *E*_*1*_ *+E*_*2*_, and the relaxation time is defined as *τ*_*0*_ = *η / E*_*1*_. Hence, the dynamic modulus in response to harmonic strain excitation of the standard solid viscoelastic model can be expressed as [21, 22]

$$E(i\omega )=\frac{E_{1}^{2}E_{2}+(E_{1}+E_{2}){\left(\omega \eta \right)}^{2}}{{(E}_{1}^{2}+{\left(\omega \eta \right)}^{2})(E_{1}+E_{2})}+i\frac{{E_{1}}^{2}\omega \eta }{{(E}_{1}^{2}+{\left(\omega \eta \right)}^{2})(E_{1}+E_{2})}.$$ (3)

Using the above viscoelastic model, the displacement-strain relation can be derived from the momentum balance of the arterial wall as

$$\rho_{s}\frac{\partial^{2}u}{\partial t^{2}}+div\;\sigma +f=0,$$ (4)

where ***u*** is wall displacement vector, ***σ*** is wall stress tensor, and ***f*** is body force vector. In order to facilitate the calculation, the above equation can be written in the cylindrical coordinate system. Since the vessel is symmetric, the radial equation of motion in a cylindrical vessel (in the absence of external body forces) with the assumption of the incompressibility of the vessel wall is given by [23]

$$\rho_{s}\frac{\partial^{2}u_{r}}{\partial t^{2}}=\frac{\partial \sigma_{rr}}{\partial r}+\frac{\sigma_{rr}-\sigma_{\theta \theta }}{r}.$$ (5)

For the cases considered in this work, this equation is subjected to the boundary condition given by

$$\sigma_{rr}{|}_{r=R_{in}\left(t\right)}=-p\left(t\right)\;\mathrm{and}\;\sigma_{rr}{|}_{r=R_{out}\left(t\right)}=0.$$ (6)

Here, *t* and *r* denote time and radial coordinates respectively, *ρ*_*s*_ is the vessel wall density, *u*_*r*_ is the radial displacement of the wall, *σ*_*rr*_ is the radial stress, *σ*_*θθ*_ is the circumferential stress, *p(t)* is the pressure, *R*_*in*_*(t)* is the inner radius of the vessel wall, and *R*_*out*_*(t)* is the outer diameter of the vessel wall. Since the arterial wall is thin and has small asymmetrical deformations, it can be treated as a membrane [21, 24]. In the thin-walled theory for the blood vessel, the axial strain can be calculated by employing Hooke’s law for an isotropic medium as

$$\varepsilon_{zz}=\frac{1}{E}(\sigma_{zz}-\mu (\sigma_{\theta \theta }+\sigma_{rr}),$$ (7)

where *μ* is the Poisson ratio. In addition, from the membrane assumption, the corresponding radial and axial stresses are

$$\left\{ \begin{array}{c} \sigma_{rr}=0\\ \sigma_{zz}=\mu \sigma_{\theta \theta } \end{array}.\right.$$ (8)

In circumferential direction, we have

$$\varepsilon_{\theta \theta }=\frac{u_{r}}{r_{0}}=\frac{1}{E}(\sigma_{\theta \theta }-{\mu \sigma }_{zz})=\frac{\sigma_{\theta \theta }}{E}(1-\mu^{2}),$$ (9)

Thus

$$\sigma_{\theta \theta }=\frac{E}{1-\mu^{2}}\frac{u_{r}}{r_{0}},$$ (10)

where *r*_*0*_ is the radius of the aorta wall before deformation, and *u*_*r*_ is the wall displacement in radial direction. It can be assumed that the flow waveform is periodic so that all variables can be expressed in terms of different harmonics of the fundamental frequency. Without loss of generality, a single harmonic of the pressure wave can be considered as the sinusoidal function given by

p(t)=Psinωt. (11)

Because of the linearity of Eqs (1) and (4), the stress and strain also have the same frequency of oscillation as the pressure. In the complex form (which greatly simplifies the analysis), the pressure, stress and strain can be written respectively as

$$p=Re(\hat{p}e^{i\omega t}),$$ (12)

$$\sigma =Re(\hat{\sigma }e^{i\omega t}),$$ (13)

$$\varepsilon =Re(\hat{\varepsilon }e^{i\omega t}).$$ (14)

Here, “Re” denotes the real part, and “^” denotes the complex amplitude. Substituting Eqs (12–14) into Eq (1) and rearranging terms gives

$$\hat{\sigma }=E\frac{1+i\omega \tau_{2}}{1+i\omega \tau_{1}}\hat{\varepsilon }=\hat{E}\hat{\varepsilon },$$ (15)

where $\hat{E}$ is the complex young’s modulus for the relaxation function, $\hat{\sigma }$ and $\hat{\varepsilon }$ are the complex stress and strain. Combining Eq (15) with wall motion Eqs (7–10), governing Eq (5) and the boundary condition (6), the displacement expression in complex form can be written as [25]

$$u=\frac{2R_{out}\;p(1+\omega^{2}\tau_{1}\tau_{2})}{E(\gamma^{2}-1)(1+\omega^{2}{\tau_{2}}^{2})}sin\omega t+\frac{2R_{out}\;p\omega (\tau_{1}-\tau_{2})}{E(\gamma^{2}-1)(1+\omega^{2}\tau_{2}^{2})}cos\omega t.$$ (16)

For the Maxwell-type standard solid viscoelastic model, we have *τ*_*1*_ = *η / E*_*1*_, *τ*_*2*_ = *η (E*_*1+*_ *E*_*2*_*) / E*_*1*_*E*_*2*_, *E* = *E*_*2*_/(*E*_*1+*_ *E*_*2*_*)*. Hence, according to Eq (16), even for the simple harmonic of the pressure waveform, there will be a complex relationship between the pressure and vessel wall displacement in the presence of the viscoelasticity. This complexity motivates the need for the development of mathematical frameworks or numerical simulations, as described in what follows, for further analyzing the problem.

### Numerical model

#### Governing equations

An arbitrary Lagrangian-Eulerian (ALE) formulation is used for the analysis of the fluid flow with moving boundaries. The ALE formulation can directly be coupled with the Lagrangian formulation in the solid domain [26]. To solve the pressure and flow fields in the fluid domain, Navier-Stokes equations for the homogenous, incompressible and Newtonian fluid are employed. For coupling the fluid-solid interface, the fundamental conditions of displacement compatibility with respect to a no-slip condition and traction equilibrium are applied as [27]

$$\vec{\nabla }∙\vec{V}=0,$$ (17)

$$\rho_{f}\left(\frac{\partial \vec{V}}{\partial t}+(\vec{V}-\vec{W})∙\vec{\nabla }\vec{V}\right)+\vec{\nabla }p=\mu \nabla^{2}\vec{V}+\vec{F_{b}},$$ (18)

$$\vec{V_{f}}=\vec{\dot{u_{s}}},$$ (19)

$$\vec{n}∙\vec{\sigma_{f}}=\vec{n}∙\vec{\sigma_{s}},$$ (20)

where $\vec{V}=(v_{y,}v_{z})$ is the flow velocity vector, *p* is the static pressure, *ρ*_*f*_ is the fluid density, *μ* is the dynamic viscosity, $\vec{W}$ is the velocity of the arbitrary Lagrangian-Eulerian (ALE) frame, $\vec{F_{b}}$ is the body force, *V*_*f*_ and $\dot{u_{s}}$ are the velocity of the fluid and structure at the interface respectively, *σ*_*f*_ is the fluid stress tensor, and *σ*_*s*_ is the solid stress tensor. For the solid domain, we consider two cases:

**Elastic wall condition**

The dynamic motion of the wall can be calculated by the Lagrangian form of the balance of the momentum equations as [28]

$$\sigma_{ij,j}+F_{i}=\rho_{s}\ddot{u_{i}},$$ (21)

where *σ*_*ij*_ is the stress tensor, *F*_*i*_ is the external force, *u*_*i*_ is the displacement vector, and *ρ*_*s*_ is the wall density. The stress tensor can be expressed as [29]

$$\sigma_{ij}=\lambda \varepsilon_{kk}\delta_{ij}+\mu (\varepsilon_{ij}+\varepsilon_{ji}),$$ (22)

where *λ* and *μ* are Lame parameters, and $\lambda =\frac{E\upsilon }{\left(1-\upsilon )(1-2\upsilon \right)},\;\mu =G=\frac{E}{2(1+\upsilon )}$. Note that the bulk modulus which describes the volumetric elasticity of the material can also be calculated by $K=\frac{E}{3(1-2\upsilon )}$.

**Fig 3:**
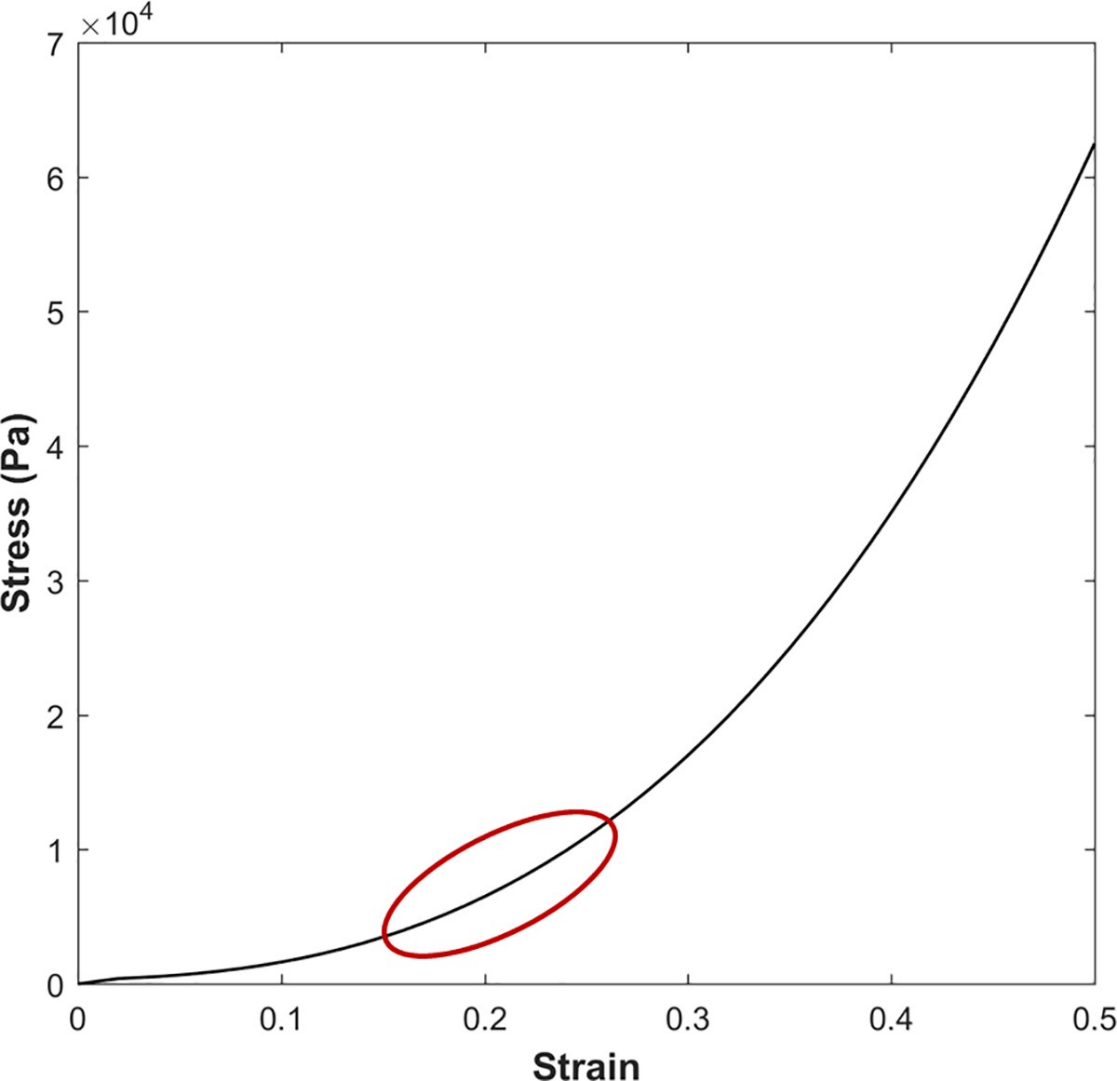
Stress-Strain data used for nonlinear case analysis. The red curve indicates the output stress-strain region.

In order to examine the vessel wall displacement in a non-linear regime, we have utilized the nonlinear elastic stress-strain data input in tabular for, extracted from Fig 3. This material model is useful for large displacement and small strain analysis.

**Viscoelastic wall condition**

Considering large deformation but small strain theory, the mechanical behavior for an inotropic and quasi-linear viscoelastic material can be expressed in tensor notation as [30]

$$S_{ij}(t)=2G(0)e_{ij}(t)+2\int_{0}^{t}e_{ij}(t-\tau )\frac{dG(\tau )}{d\tau }d\tau ,$$ (23)

$$\sigma_{kk}(t)=3K(0)\varepsilon_{kk}(t)+3\int_{0}^{t}\varepsilon_{kk}(t-\tau )\frac{dK(\tau )}{d\tau }d\tau ,$$ (24)

where *t* is the time, $S_{ij}=\sigma_{ij}-\frac{1}{3}\delta_{ij}\sigma_{kk}$ is the deviatoric stress, *σ*_*ij*_ is the stress, *δ*_*ij*_ is the Kronecker delta, $e_{ij}=\varepsilon_{ij}-\frac{1}{3}\delta_{ij}\varepsilon_{kk}$ is the deviatoric strain, *ε*_*ij*_ is the strain, and *G(t)* and *K(t)* are shear and bulk modulus respectively. To solve the above convolution integrals, the full-time history of the deformation and material variables must be considered—a challenging task. Alternatively, we can characterize the viscoelastic behavior of the material as relaxation functions that are described by exponential series [31]. This method takes advantage of recursive equations for evaluation hereditary integrals (Eqs (23 and 24)). Considering the generalized Maxwell model, we can express the shear and bulk modulus by Prony series expansions as

$$G(t)=G_{\infty }+\sum_{i=1}^{N}G_{i}e^{-\frac{t}{\vartheta_{i}}},$$ (25)

$$K(t)=K_{\infty }+\sum_{i=1}^{N}K_{i}e^{-\frac{t}{\gamma_{i}}},$$ (26)

where *N* is the number of chains in the Maxwell model, *G*_∞_ and *K*_∞_ are the steady-state shear and bulk modulus respectively, *G*_*i*_ and *K*_*i*_ are the coefficients, and *ϑ*_*i*_ and *γ*_*i*_ are the relaxation times in the Prony series expansions. Since we are using a standard viscoelastic model in this study, we consider only the first chain (*N* = 1) in the Prony series for the material properties. With the viscoelastic material properties in hand (evaluated by Eqs (25 and 26)), we can numerically compute the discretized convolution integrals of Eqs (23) and (24).

#### Boundary conditions

At the inlet, a physiological flow waveform (similar to [19, 32]) with a flat velocity profile is imposed and scaled to give a cardiac output (CO) of 4.6 L/min. In the fluid domain, a constant normal traction of P_end_ = 10.5mmHg is imposed at the end of outflow boundary model, while in the solid domain, zero normal traction is imposed on the outer surface of the straight vessel and outflow boundary [19]. To simulate the effect of truncated vasculature, an extension tube boundary model was used for the outflow boundary condition at the terminal of the abdominal aorta. This boundary model extends the computational model with a straight elastic tube connected to a contracted rigid tube. The parameters of the outflow boundary condition model are given in Table 1, where the contraction ratio is the ratio of the radius of the rigid boundary tube (after the contraction) to the original radius (before the contraction) [33]. In all simulations, CO, the parameters of the outflow boundary condition, and the shape of the inflow wave were kept constant.

#### Wave intensity analysis

WIA is based on the method of characteristics which has been used in analogous problems in gas dynamics. In this method, the hyperbolic system of one-dimensional equations of motion in *z* and *t* is transformed into a system of ordinary differential equations along the two families of the characteristics defined as $\frac{dz}{dt}=U\pm c$, where *U* is the average velocity over the cross section and $c=\sqrt{\frac{A}{\rho \frac{\partial A}{\partial P}}}$ is the speed of propagation [5]. Following an impermeable assumption for the wall, the Riemann invariants can be defined on the characteristic lines as

$$dR_{\pm }=dU\pm \frac{1}{\rho c}dP.$$ (27)

Solving for *dP* and *dU*, their corresponding product can be derived as

$$dI=dPdU=\frac{\rho c}{4}(dR_{+}^{2}-dR_{-}^{2}),$$ (28)

where d*I* is defined as the WI in units of power per unit area (e.g. W/m^2^). Based on Eq (28), the contribution of the forward running wave (d*R*_+_) to the WI is always positive, while the contribution of the backward running wave (d*R*_−_) to the WI is always negative. Therefore, if d*I*>0, the forward waves are dominant; and if d*I* <0, the backwards (reflected) waves are dominant. This allows one to easily determine wave propagation at each time. If the incremental waves are assumed to be additive, the WI can be further decomposed into purely forward (+) and purely reflected (-) waves [34] as

$$dI_{+}=\frac{1}{4\rho c}{\left(dP+\rho cdU\right)}^{2},$$ (29)

$$dI_{-}=\frac{-1}{4\rho c}{\left(dP-\rho cdU\right)}^{2}.$$ (30)

As can be noted from Eqs (29) and (30), simultaneous increments of pressure and flow (velocity) are needed to compute WI. Our focus in this study is to compare the pressure-based WI waveform (obtained based on the pressure and velocity) with the displacement-based WI (obtained from velocity and vessel wall displacement). Based on Eq (16), we can rewrite the wall displacement as

$$D=\Delta R=\hat{R}sin(\omega t+\varphi ),$$ (31)

where $\hat{R}$ is the amplitude for the vessel wall displacement, and *φ* is the phase difference between the vessel wall displacement and pressure harmonic. We can further assume that vessel wall displacement amplitude $\left(\hat{R}\right)$ and pressure inside the tube (*P*) are correlated by the proportionality coefficient in the short time interval (which depends on the wall modulus of elasticity, inner and outer diameter, and viscoelasticity parameter of the wall (*τ*_1_,*τ*_2_)). Hence, we can introduce an alternative parameter similar to the WI, defined to be d*I*_*D*_, which is the product of the increment in diameter and the flow given by

$$dI_{D}=\frac{1}{\hat{k}}dD\times dU,$$ (32)

where $\hat{k}$ is the proportionality coefficient. In order to compare the above parameter with the WI that is defined based on the pressure and flow, we can further non-dimensionalize these two parameters by dividing each one over the maximum value in the cycle. From this, we can compare the waveform obtained based on these two variables at each site. The non-dimensionalized variables are denoted with asterisks: DI*_D_ for dimensionless displacement-based WI and DI*_P_ for dimensionless pressure-based WI.

#### Computational model

In order to solve the equations of solids and fluids numerically, an ALE finite element method with the two-way coupling scheme of fluid-structure interaction is employed at each time step [29]. The fluid domain is modeled with 17,416 total number of four-node 3D axisymmetric elements, while the solid domain is modeled with 2,420 total number of nine-node 2D axisymmetric elements. The linearized equation of the coupled system was solved with the direct or non-iterative solution technique [35]. A Newton-Raphson iteration scheme was used for the time integration with a time step of 0.00125s. Further simulations with different time steps and grid sizes confirm that these results are independent of spatial and temporal discretization. For the same spatial discretization, the pressure wave solutions for time steps of 0.000625s and 0.00125s reveal a maximum relative error at the middle of the straight vessel of less than 3%. Similarly, for the same time step, doubling the number of spatial elements reveals a maximum relative error of less than 3%. The commercial package ADINA 9.4 (ADINA R&D, Inc., MA) is used to run all simulations. Each simulation is started at rest with no loading condition, and the simulation is carried out to at least 10 cycles in order to ensure that the mean pressure reaches an oscillating steady state condition.

## Results

### Regular vessel wall condition

#### Elastic wall condition

Based on Eq (31), *φ* is the phase difference between the propagated pressure and vessel wall displacement (which depends on the wall material behavior). Utilizing the standard viscoelastic wall model introduced in Eq (5), the vessel wall displacement in response to a harmonic of pressure was described in Eq (16). Therefore, the phase difference between the pressure and vessel wall displacement can be given by

$$\varphi =tan\left(\frac{\omega (\tau_{1}-\tau_{2})}{\left(1+\omega^{2}\tau_{1}\tau_{2}\right)}\right)$$ (33)

All the parameters on the right-hand side of Eq (28) are real and positive values. However, note that Eq (33) is derived for a single pressure harmonic in a linear standard viscoelastic case.

**Fig 4:**
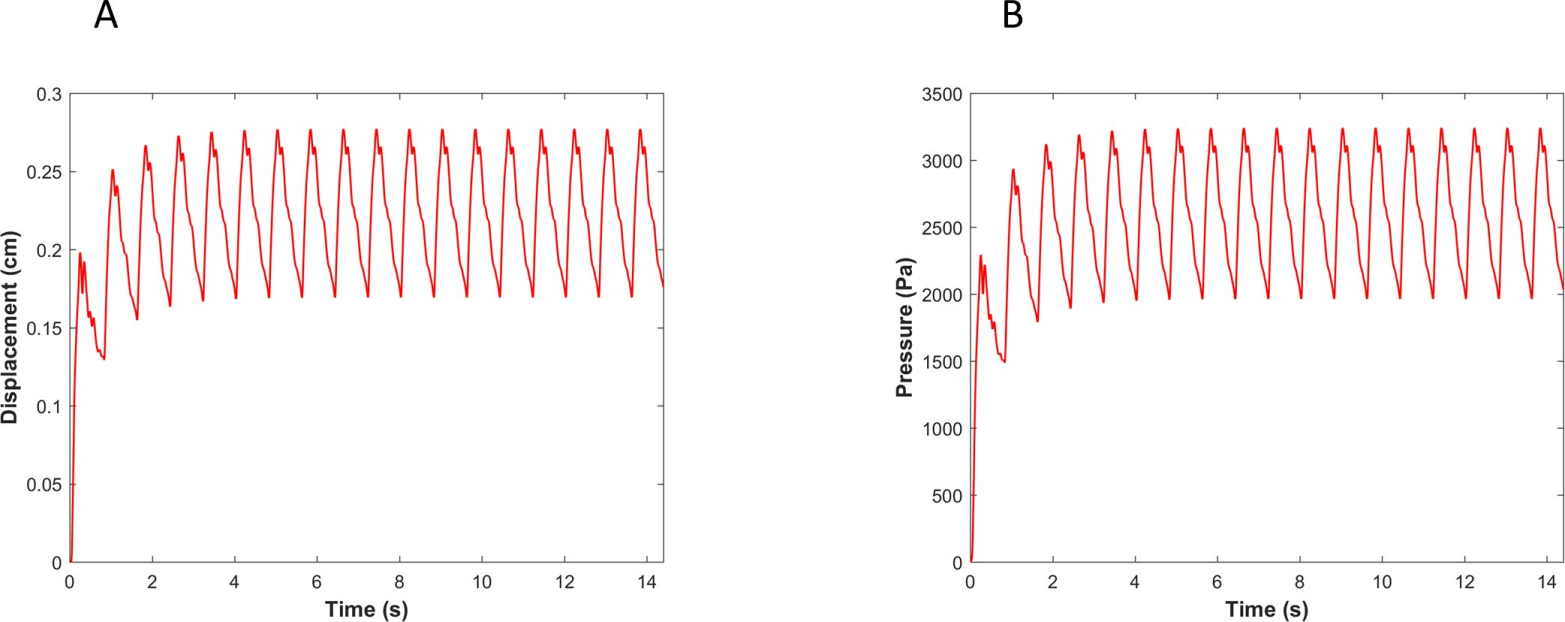
(A) The vessel wall displacement and (B) pressure reaches a steady state condition in elastic wall condition.

**Fig 5:**
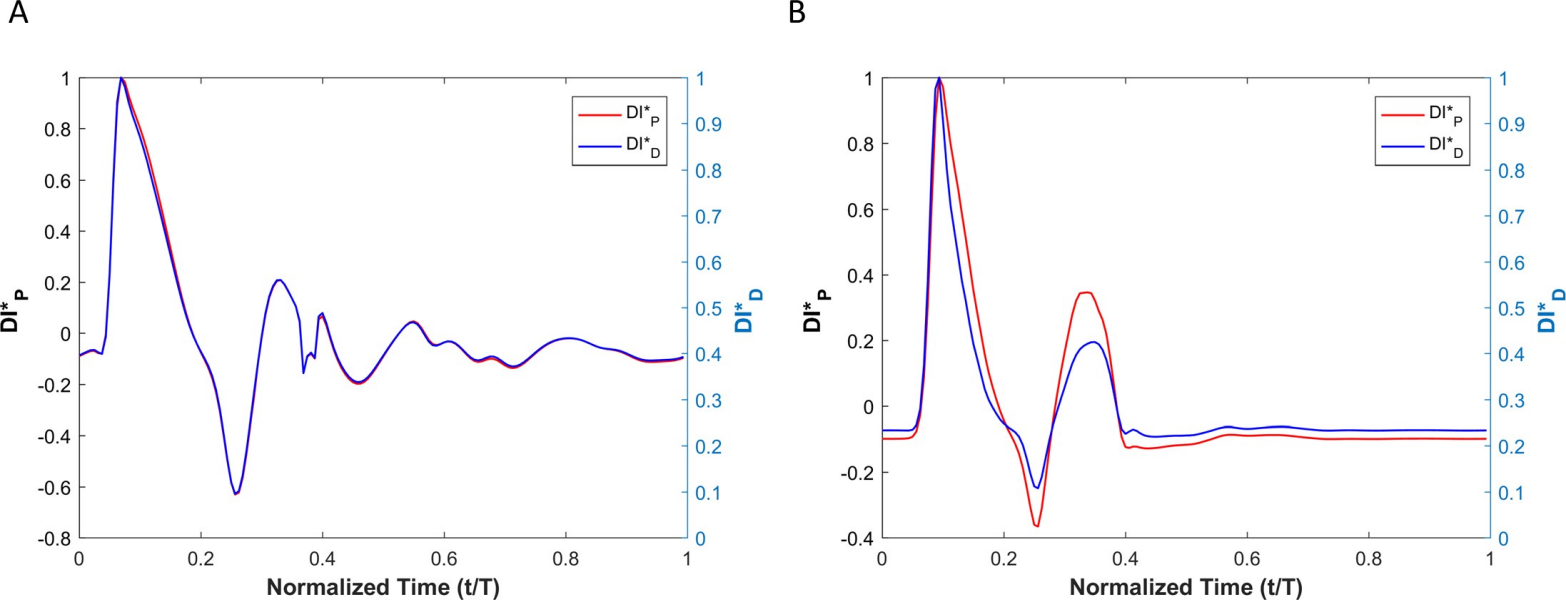
Comparison of the WI that is defined based on the pressure versus the one that is defined based on the diameter for (A) a linear elastic wall, and (B) a nonlinear hyper elastic wall conditions. DI*_D_ is the dimensionless displacement-based WI and DI*_P_ is the dimensionless pressure-based WI (see the text for details).

It is not clear if different pressure harmonics interact linearly with each other in a viscoelastic wall under physiological conditions. Therefore, we compared the pressure-based WI (invasive in practice) with displacement-based WI (non-invasive in practice) numerically using the CFD-FSI model described in method section. Fig 4 shows the pressure and the vessel wall displacement waveforms from of our numerical simulation that demonstrates convergence to a steady-state oscillatory condition. WI results for the linear and nonlinear elastic cases are demonstrated in Fig 5.

**Fig 6:**
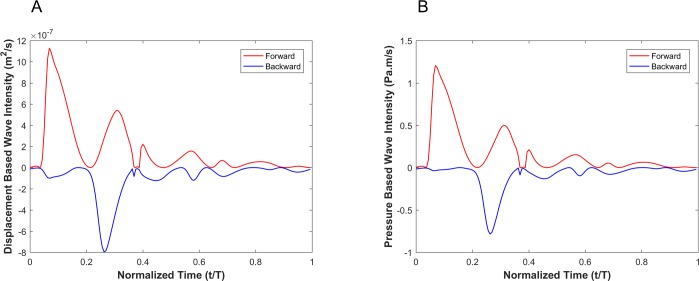
Forward (red) and backward (blue) running components of the wave intensity for the displacement-based wave intensity (A) and pressure-based wave intensity (B) under the elastic wall condition.

Fig 6 shows the decomposition of both the displacement-based and pressure-based wave intensities into forward and backward running waves (see Eq (28)). Since the goal is to compare the shape of the displacement-based and the pressure-based wave intensity components, we have not scaled the forward and backward running waves and hence report their absolute amplitudes.

#### Viscoelastic wall condition

**Fig 7:**
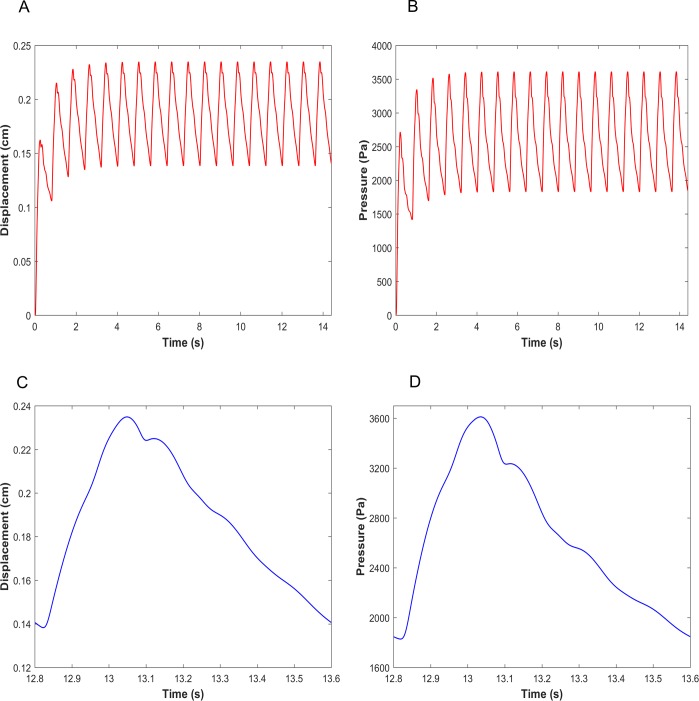
(A) Vessel wall displacement and (B) pressure reach steady state conditions in viscoelastic wall conditions. Steady state (C) Vessel wall displacement and (D) pressure during one cardiac cycle.

Fig 7 demonstrates the pressure and the vessel wall displacement waveforms from of our numerical simulation of a viscoelastic wall that demonstrates convergence to a steady-state oscillatory condition. As shown in this figure, there are clear differences between the extracted vessel wall displacement profile and the pressure profile (e.g., the relative height of the dicrotic notch).

**Fig 8:**
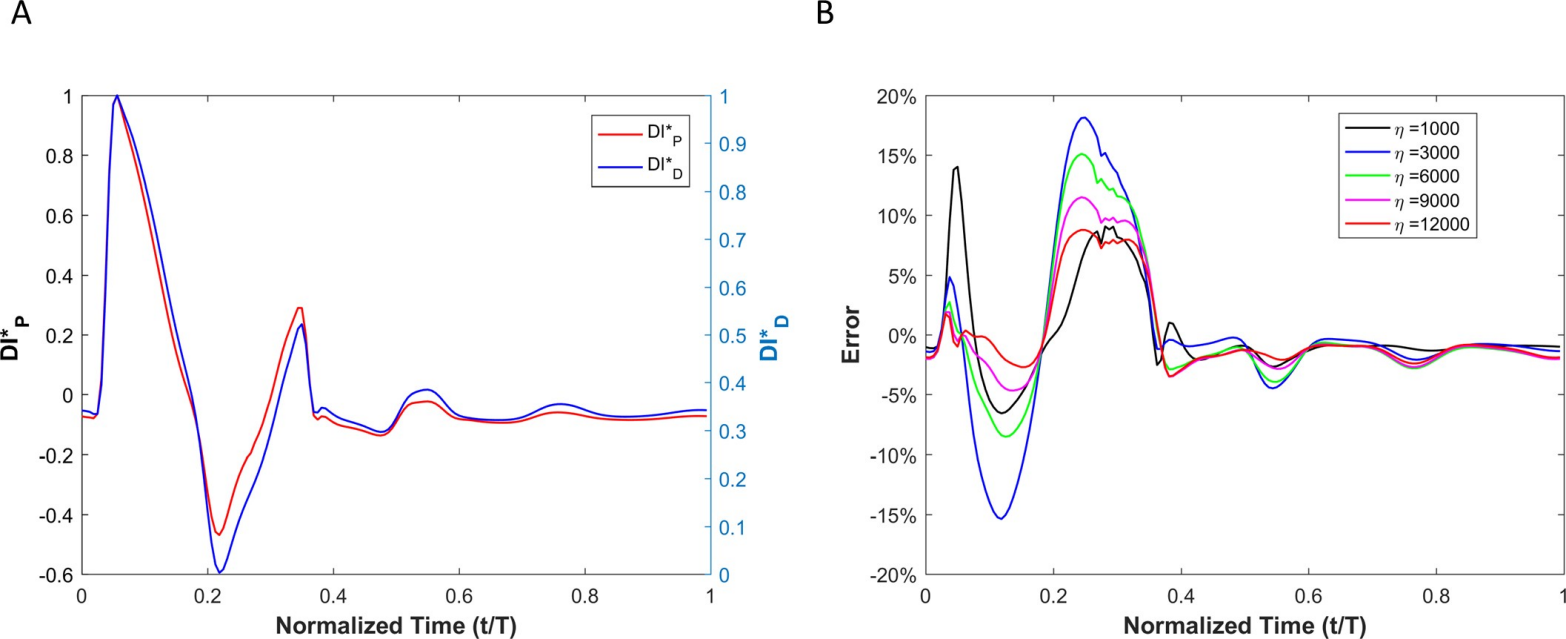
(A) Comparison of the WI that is defined based on the pressure versus the one that is defined based on the diameter for viscoelastic wall condition. (B) The calculated error between the two wave intensities for different vessel wall viscosities.

WI results for viscoelastic vessel wall condition cases are demonstrated in Fig 8. Results of different wall viscosities (within the physiological range) are demonstrated in Fig 8B. The root mean square error (RMSE) for all cases (wall viscosities: 1000Pa·s, 3000Pa·s, 6000Pa·s, 9000Pa·s and 12000Pa·s) are shown in Table 2. Note that the RMSE is defined as $RMSE=\sqrt{\frac{1}{n}\sum_{t=1}^{n}{e_{t}}^{2}}$, where *n* is number of points within a cardiac cycle and $e_{t}=|DI_{D}^{*}-DI_{P}^{*}|$.

**Table 2:** RMSE and the peak ratios for the regular vessel wall.

Straight Vessel					
*η (Pa·s)*	1000	3000	6000	9000	12000
RMSE	0.0380	0.0682	0.0545	0.0421	0.0333
PeakRD+	1.0073	1.0087	1.0331	1.0239	1.0199
PeakRD-	-1.8023	-1.6685	-1.6796	-1.7014	-1.7250
PeakRP+	1.0034	1.0384	1.0301	1.0297	1.0305
PeakRP-	-1.9753	-2.2265	-2.1322	-2.0420	-1.9811

Ratios of WI peaks are demonstrated in Table 2. The 'peakRD^+^' is defined as the ratio of the first positive peak over the second positive peak for the displacement-based WI. The 'peakRD^-^' is defined as the ratio of the first positive peak to the first negative peak for the displacement-based WI. The 'peakPD^+^' is defined as the ratio of the first positive peak to the second positive peak for the pressure-based WI. The 'peakPD^-^' is defined as the ratio of the first positive peak to the first negative peak for pressure-based WI.

**Fig 9:**
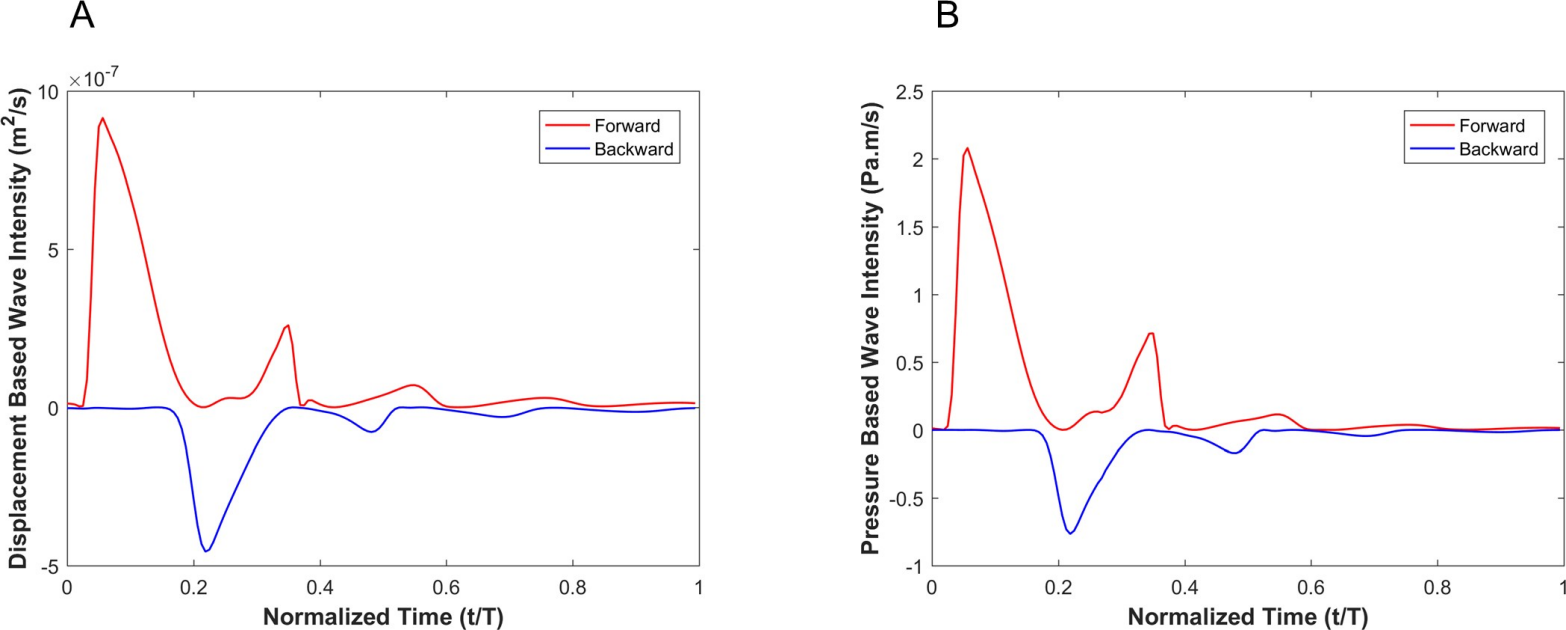
Forward (red) and backward (blue) running components of the wave intensity for the displacement-based wave intensity (A) and pressure-based wave intensity (B) under the viscoelastic wall condition with wall viscosity of 6000 Pa.s.

Fig 9 shows the decomposition of the displacement-based and pressure-based wave intensities into forward and backward running waves for the viscoelastic wall condition with a wall viscosity of 6000Pa·s.

### Irregular vessel wall condition

#### Elastic wall condition

**Fig 10:**
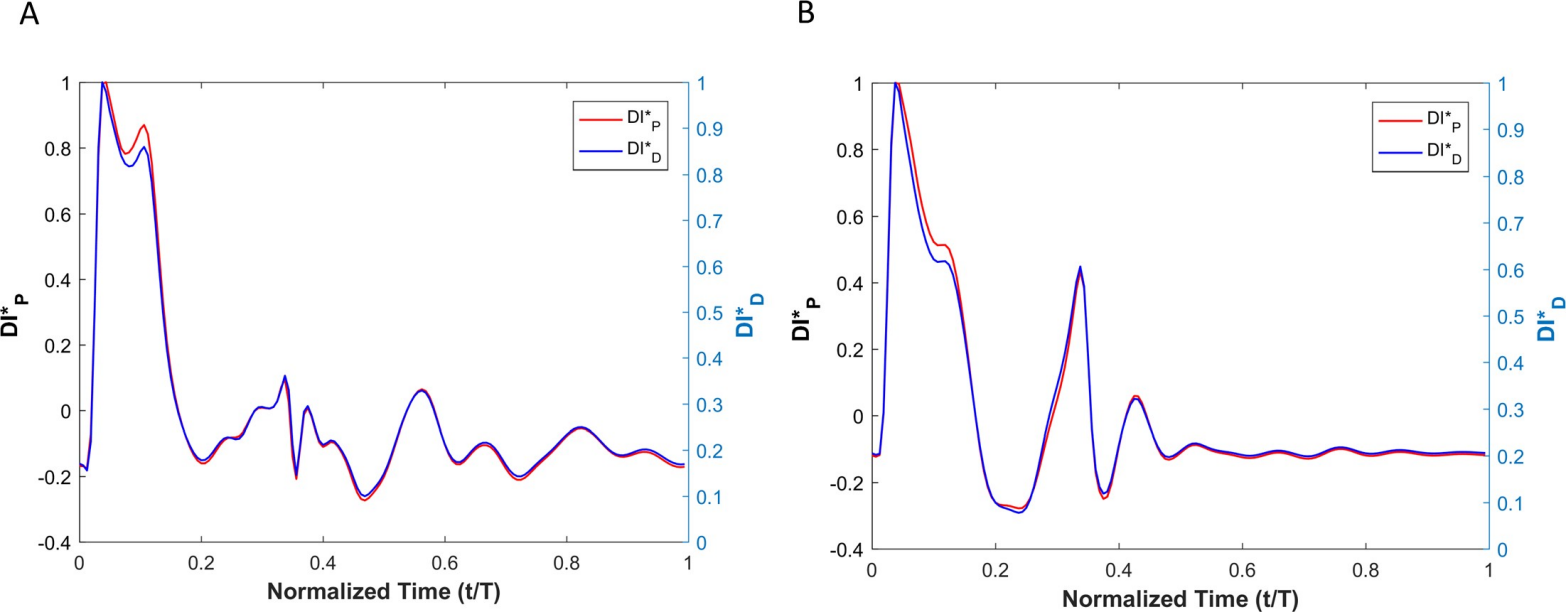
Comparison of the WI that is defined based on the pressure versus the one that is defined based on the diameter for elastic wall condition for (A) aneurysm, and (B) stenosis cases.

Fig 10 shows the results at point A and point B (see Fig 2) in a linear elastic vessel wall with downstream irregular geometric features in the form of an aneurysm (Fig 10A) or a stenosis (Fig 10B).

#### Viscoelastic wall condition

**Fig 11:**
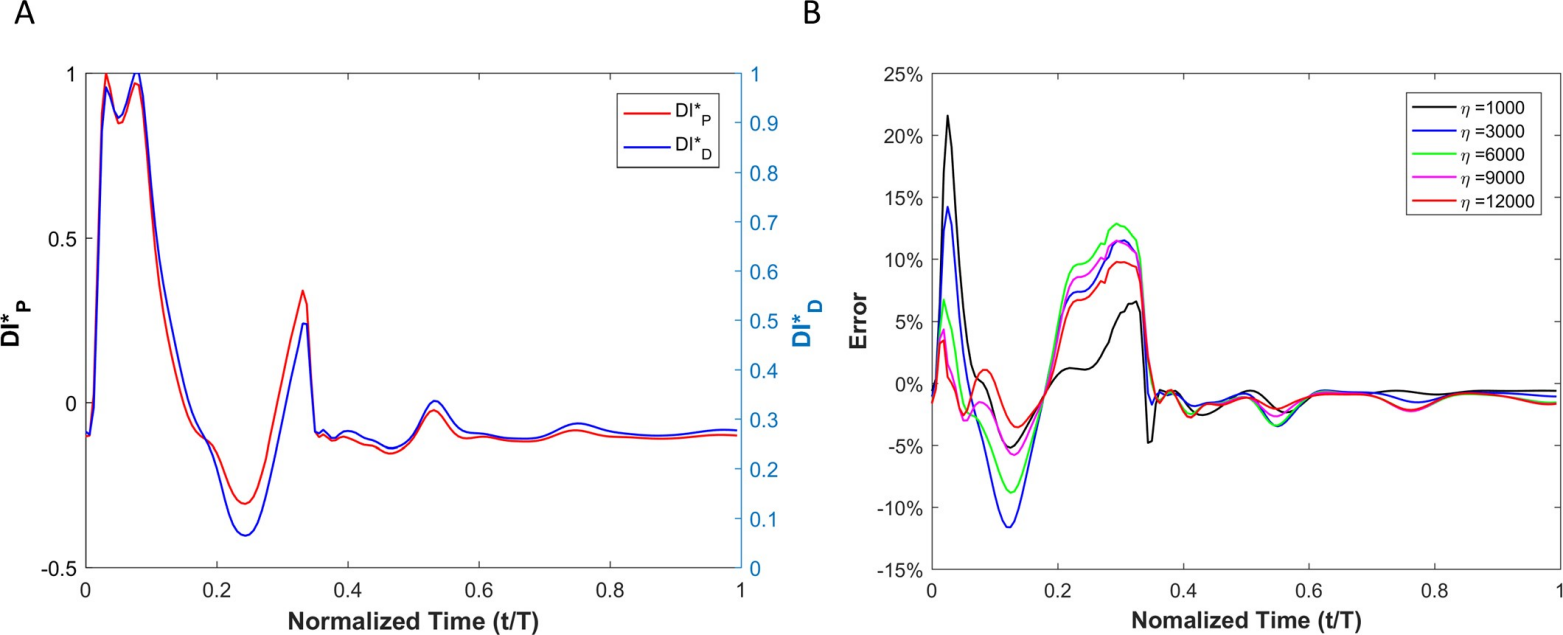
(A) Comparison of the WI that is defined based on the pressure versus the one that is defined based on the diameter for viscoelastic wall condition in the aneurysm case. (B) Calculated error between the two wave intensities for different vessel wall viscosities.

Results of displacement-based WI ($DI_{D}^{*}$) and pressure-based WI ($DI_{P}^{*}$) of a viscoelastic aneurysmal vessel is shown Fig 11A. The point-by-point absolute error (*e*_*t*_) between $DI_{D}^{*}$ and $DI_{P}^{*}$ at different levels of wall viscosity (1000 *Pa·s* <*η<*12000 *Pa·s*) during a complete cardiac cycle is depicted in Fig 11B.

**Fig 12:**
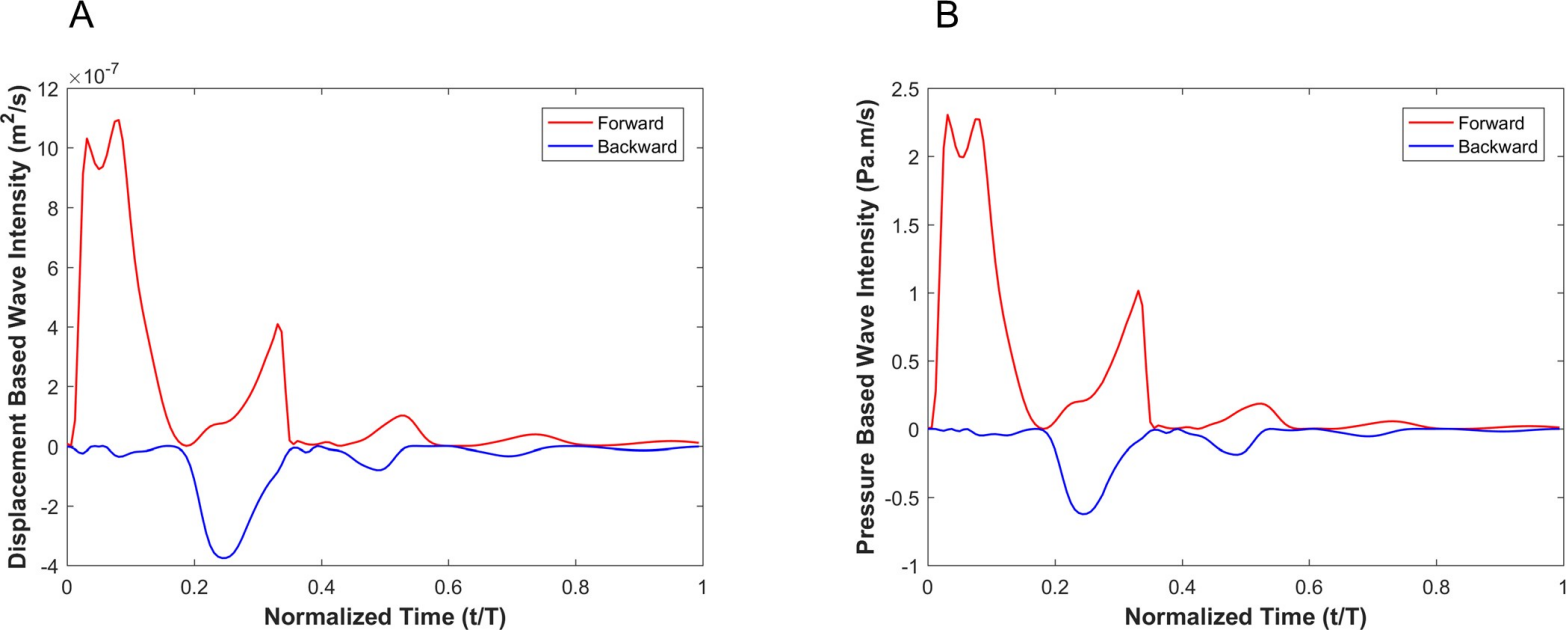
Forward (red) and backward (blue) running components of the wave intensity for the displacement-based wave intensity (A) and pressure-based wave intensity (B) under the viscoelastic wall condition with wall viscosity of 6000 Pa.s in the presence of aneurysm.

In the presence of an aneurysm, Fig 12 shows the decomposition of the displacement-based and pressure-based wave intensities into forward and backward running waves for the viscoelastic wall condition with a wall viscosity of 6000Pa·s.

**Fig 13:**
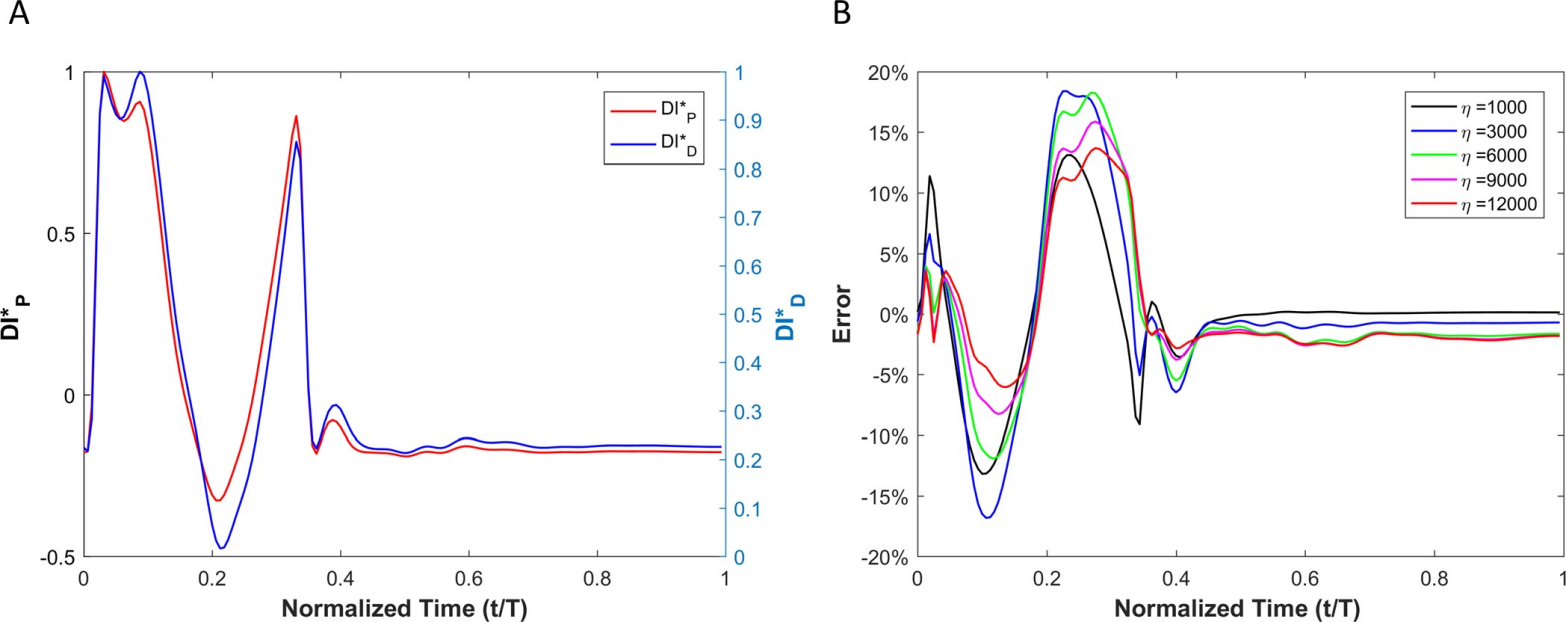
(A) Comparison of the WI that is defined based on the pressure versus the one that is defined based on the diameter for viscoelastic wall conditions in the stenosis case. (B) Calculated error between the two wave intensities for different vessel wall viscosities.

Fig 13A shows the results of $DI_{D}^{*}$ and ($DI_{P}^{*}$) for a viscoelastic stenotic vessel. Fig 13B demonstrates the point-by-point error (*e*_*t*_) between $DI_{D}^{*}$ and $DI_{P}^{*}$ at different levels of wall viscosity (1000 *Pa·s* <*η<*12000 *Pa·s*) during a complete cycle.

**Fig 14:**
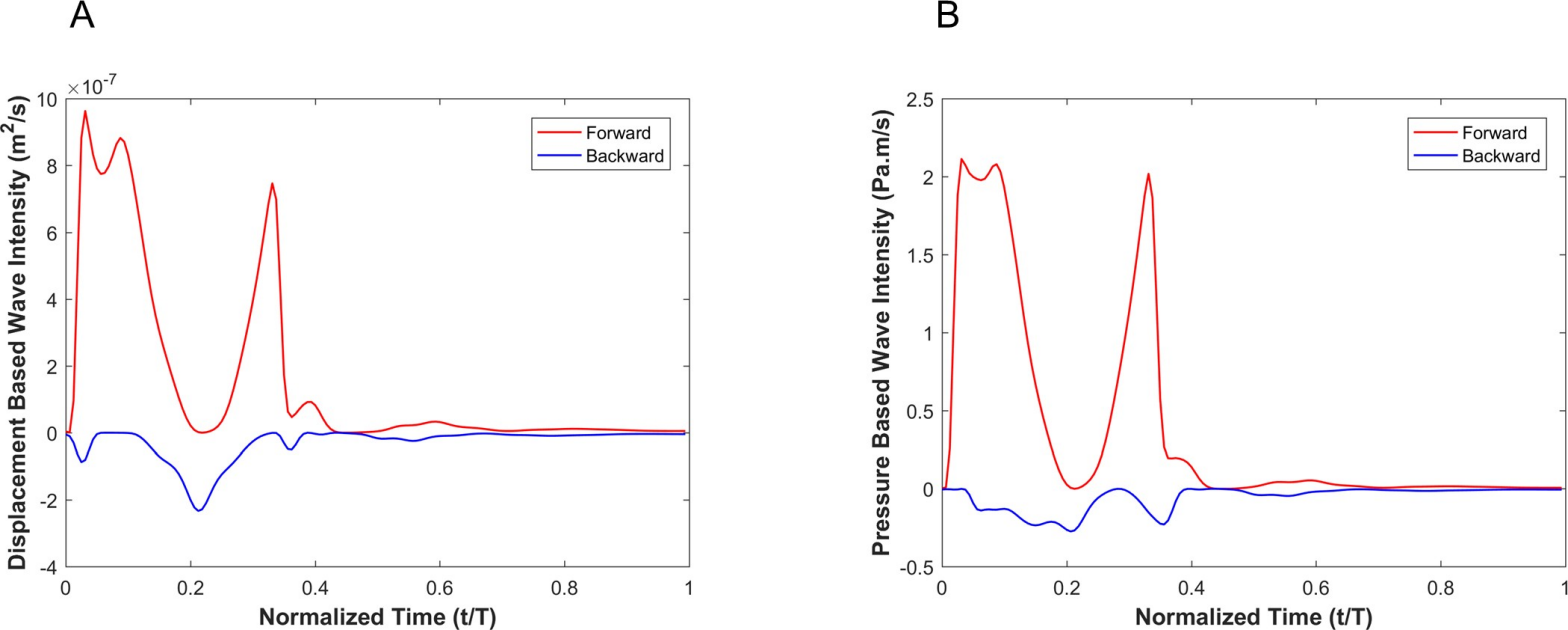
Forward (red) and backward (blue) running components of the wave intensity for the displacement-based wave intensity (A) and pressure-based wave intensity (B) under the viscoelastic wall condition with wall viscosity of 6000 Pa.s in the presence of stenosis.

In the presence of stenosis, Fig 14 shows the decomposition of the displacement-based and pressure-based wave intensities into forward and backward running waves for the viscoelastic wall condition with a wall viscosity of 6000Pa·s.

The RMSE (between $DI_{D}^{*}$ and $DI_{P}^{*}$) over the full cardiac cycle and the different peak ratios of WI waveforms for all viscoelastic vessels with geometrical irregularities—aneurysm or stenosis—are provided in Table 3.

**Table 3:** RMSE and the peak ratios for the irregular vessel wall in the presence of aneurysm and stenosis.

**Aneurysm**					
*η(Pa·s)*	1000	3000	6000	9000	12000
RMSE	0.0370	0.0489	0.0473	0.0401	0.0330
PeakRD+	1.0148	1.0083	1.0002	1.0049	1.0116
PeakRD-	-4.4166	-2.9432	-2.4772	-2.3684	-2.3613
PeakRP+	1.0124	1.0459	1.0319	1.0155	1.0039
PeakRP-	-4.6529	-3.7658	-3.2592	-2.9796	-2.8126
**Stenosis**					
*η(Pa·s)*	1000	3000	6000	9000	12000
RMSE	0.0515	0.0713	0.0674	0.0573	0.0493
PeakRD+	1.0018	1.0135	1.0132	1.0014	1.0071
PeakRD-	-2.1433	-2.1022	-2.1041	-2.1176	-2.1430
PeakRP+	1.0113	1.0245	1.0257	1.0251	1.0243
PeakRP-	-2.8815	-3.2327	-3.0631	-2.8503	-2.7260

## Discussion

In this study, we examined the impact of vessel wall nonlinearity and viscoelasticity on the accuracy of the displacement-based WI (which can be measured non-invasively in clinical practice using imaging modalities) compared with the original pressure-based formulation of WI (which requires invasive measurement in clinical practice). We have used computational vessel models utilizing an Arbitrary Lagrangian-Eulerian Finite Element method in order to facilitate our study. It has been shown recently by Segers et al. [36] that wave reflections play a prominent role in conducting wave analysis and determining the characteristic parameters (such as pulse wave velocity) from the combination of pressure, velocity, or cross-sectional waveforms. To account for the effects of wave reflection, an extension tube boundary model has been utilized in our simulations to capture the compliance, resistance, and wave reflections of the downstream vasculature.

The error associated with displacement-based WI (compared with the pressure-based WI) is the result of the phase difference (*φ*) between the pressure and vessel wall dilation. Based on the theoretical analysis of a single pressure harmonic (Eq 33), it can be concluded that the effect of viscoelastic behavior of the vessel wall is not considerable as long as viscoelastic time relaxation coefficients (*τ*_1_ and *τ*_2_) are negligible. Since the dependency of *φ* on *τ*_i_ (i = 1 and 2) is nonlinear (see Eq 33), there will be a subsequent nonlinear and complex relationship between the error of $DI_{D}^{*}$ and the degree of viscoelasticity (e.g. *η*) when *τ*_i_ (i = 1 and 2) become considerable.

Results from numerical simulations of a linearly elastic vessel wall demonstrate that the pressure-based WI (DI*_P_) is identical to displacement-based WI (DI*_D_). As shown in Fig 5 for nonlinear and non-viscoelastic wall vessels, there is no phase lead or lag between DI*_P_ and DI*_D_. However, the WI peak ratios of DI*_P_ and DI*_D_ are different in vessels with nonlinear stress-strain relationships (even in the absence of viscoelasticity). Results of vessels with viscoelastic wall properties, on the other hand, demonstrate that there is a phase difference between the DI*_P_ and DI*_D_ waveforms (Fig 8A). This phase difference results in errors between DI*_P_ and DI*_D_ (*e*_*t*_), as shown in Fig 8B. In general, the error grows with increasing *η*. However, the error displays a nonlinear dependency with increasing viscosity (see Fig 8B and Table 2) as predicted by the aforementioned analytical model. In Table 2, the errors between DI*_P_ and DI*_D_ waveforms for different viscoelastic cases are reported as RMSE. The results from our straight viscoelastic vessel wall model suggest that the maximum corresponding error associated with displacement-based WI is bounded around 6.8%. One of the main advantages of WIA is the ability to decompose the wave at any time and location into its forward and backward running components (see Eq (28)). Forward waves in the vasculature are mostly caused by the heart, and backward waves are the result of reflections. This decomposition reveals more aspects of the wave dynamics when the forward and backward waves are of similar magnitude (so that the net wave intensity is small even though the waves can be large). The results from Figs 6 and 9 show that the backward and forward waves from displacement-based WI in elastic and viscoelastic cases have the same features as the backward and forward waves from a pressure-based WI.

The timings of WI peaks contain information about the physics of waves in the vascular network and provide insight into the underlying cardiovascular pathology and pathophysiology [10, 37–39]. For example, in the case of aortic WI analysis, the first peak corresponds to the initial compression caused by a left ventricle contraction (positive peak), the second peak corresponds to the reflection of the initial contraction (negative peak), and the third peak corresponds to the dominant forward wave at the end of systole (which is again positive). Therefore, it is of great clinical importance to be aware of the order of the non-invasive displacement-based WI error in capturing peaks. As shown in Table 2, the peak ratio for DI*_P_ and DI*_D_ are similar. However, the error associated with the ratio of the first positive peak to the second positive peak is much smaller than the error in the ratio of the first positive peak to the first negative peak.

In order to encourage more widespread deployment of displacement-based WIA, we considered two types of vascular disease-related geometrical irregularities: aneurysm and stenosis. These two cases impose opposite effects on wave reflection; an aneurysm act as an open-end reflection site, but stenosis operates as a closed-end reflection site. The first type, an aneurysm, is defined as a permanent localized dilation of a vessel (similar in dimension to an adult human aorta) with at least a 50 percent increase in the diameter compared with the expected normal diameter [40]. Although most aneurysms are asymptomatic, they are observable with MRI or other non-invasive measurement techniques [41]. MRI or echo imaging of an aneurysm provides information about the size, but it does not provide reliable information about the stability or rupture risk of an aneurysm. Recent studies have shown that the pulsatile hemodynamics play a significant role in risk evaluation of an aneurysm [42, 43]. The second type of irregularity considered, a stenosis, is a localized narrowing in the arterial lumen that is typically the result of atherosclerosis [44]. Our analysis shows that the DI*_P_ and DI*_D_ waveforms are almost identical if the vessel wall is purely elastic, though there exist negligible differences that might be associated with simplifications in the numerical model. Similarly to the straight vessel condition, there is a phase difference and lag effect between the DI*_P_ and DI*_D_ for viscoelastic cases as demonstrated in Figs 11 and 13. As the viscous effect increases (η increases), the error will grow in general. However, the error shows nonlinear fluctuation with increasing viscosity (see Fig 11, Fig 13 and Table 3). Similar to the straight vessel case, this is also in agreement with the analytical model of Eq (33). As demonstrated in Table 3 for the viscoelastic vessels with irregular wall conditions, the maximum corresponding error associated with displacement-based WI is around 7.13%. In addition, for both the aneurysm and stenosis cases, the displacement-based WI and the pressure-based WI have been decomposed into their forward and backward running components (Figs 12 and 14). For the aneurysm case, the shape of the forward and backward running components is almost identical for the displacement-based and pressure-based wave intensities. For the stenosis case, although the shape of the forward running wave is almost identical for the displacement-based and pressure-based wave intensities, there is some disagreement for the backward running waves between the displacement-based and pressure-based wave intensities. This can be attributed to the nature of stenotic irregularities, resulting in the presence of the closed-end reflection site in the vessel. However, the effect of this disagreement on the total magnitude of the measured WI is not significant for the displacement-based and pressure-based wave intensities. Lastly, it should be noted that similarly to the straight viscoelastic vessel case, the error associated with the ratio of the first positive peak over the second positive peak (between the DI*_P_ and DI*_D_) is much smaller than the error in the ratio of the first positive peak over the first negative peak (see Table 3).

So far, WIA has expanded our knowledge in arterial physiology and pathophysiology in the systemic circulation [45], cerebral vasometer tone [46], and pulmonary circulation [47]. Lots of effort has been made regarding the coronary WIA [48], myocardial infarction [49] and ischaemic heart disease [50]. In addition, the WI decomposition into the forward running and backward running waves has provided further information into cardiovascular physiology and the direction of the blood into the coronary arteries [51]. However, the current requirement of having invasive measurements of pressure and flow is a limitation for WI applications. The systematic research work conducted here provides a fundamental groundwork for expanding the usage of the non-invasive displacement-based WIA technique into the broader clinical environment.

## Limitation of the study

With regards to the CFD model, blood was assumed to be an incompressible Newtonian fluid and the truncated vasculature was modelled with an extension tube boundary model. These are reasonable assumptions in that they don’t affect the conclusion of this manuscript. Regarding the boundary conditions, we have imposed a flat velocity profile at the inlet of the straight vessel to simulate the pulsatile flow behavior in the system. Although the fluid in this study is a viscous flow, the flat velocity profile has more features in common with the entry flow regime than the fully developed parabolic flow profile. In addition, as the wave characteristics in this study are measured far from the inlet, the influence of the flow profile beyond the inlet boundary is negligible. Hence, this boundary condition should provide accurate results for this study. Lastly, in this study, we have only considered the simplified geometry of straight vessels in and, therefore, the curvatures and bifurcations have been excluded. The complexity of the geometry may provide subtle changes of the pressure and diameter wave; however, this will have minimal impact on the behavior that has led to the overall conclusion of our findings.

## Conclusion

Our results suggest that for large arteries demonstrating linear elastic behavior (such as the carotid artery or the aorta), the displacement-based WI is almost identical to the pressure-based WI. This is clinically significant since displacement-based WI can be measured non-invasively (as opposed to the pressure-based WI, which requires invasive measurement techniques). Our results indicate that the existence of vessel wall abnormalities such as an aneurysm or stenosis does not impact the accuracy of displacement-based wave intensity. Our results additionally indicate that wall viscoelasticity creates a phase difference between pressure and vessel wall dilation that results in a deviation of displacement-based WI when compared with pressure-based WI. This is the source of error between DI*_P_ and DI*_D_ and, as predicted by the analytical model, this error increases nonlinearly with increasing viscosity. However, at maximum, this viscoelasticity error is only 6.8% for the regular vessel wall (straight) and 7.13% for the irregular vessel wall (in the presence of aneurysm and stenosis) for the cases considered in this manuscript. In addition, our results show that the error associated with the ratio of the first positive peak over the second positive peak (between the DI*_P_ and DI*_D_) is much smaller than the error in the ratio of the first positive peak over the first negative peak for both regular and irregular vessel cases. Hence the results of this study ultimately suggest that, although utilizing displacement-based WI introduces errors into WI analysis for vessels with viscoelastic behavior (which mainly exist in only small-size vessels), these errors are small and are an acceptable compromise in order to enable measurements of WI *non-invasively* (which is of great clinical importance).

## Supporting information

S1 File Raw simulation data for wave intensity analysis. (ZIP)
